# Clinical course and visual outcome in patients with diabetes mellitus and uveitis

**DOI:** 10.1186/1756-0500-6-167

**Published:** 2013-04-29

**Authors:** Kadambari S Oswal, Ramesh R Sivaraj, Philip I Murray, Panagiota Stavrou

**Affiliations:** 1 Birmingham and Midland Eye Centre, Birmingham, UK; 2 Academic Unit of Ophthalmology, University of Birmingham, Birmingham, UK; 3 Academic Unit of Ophthalmology, Birmingham and Midland Eye Centre, City Hospital, Birmingham B18 7QU, UK

**Keywords:** Diabetes mellitus, Diabetic retinopathy, Glycaemic control, Uveitis, Visual outcome

## Abstract

**Purpose:**

We report the clinical course and visual outcome of patients with diabetes mellitus (DM) who subsequently developed uveitis from any cause.

**Methods:**

Longitudinal, retrospective case note review.

**Results:**

A total of 36 patients (M/F: 18/18, 58 eyes) were included, Of the 36 patients, 35 had Type 2 DM and one had Type 1 DM. Mean age of onset of DM was 49 years and uveitis 55 years. The uveitis was bilateral in 22 (61%) patients. There were 19 patients with anterior uveitis, 12 with panuveitis and 5 with intermediate uveitis. Mean follow up was 4.4 years (range 1-18). Mean number of uveitis recurrences was 3 (range 1-7). Causes of vision of 6/18 or worse appeared related to the uveitis in 9 eyes and diabetes in 4 eyes. Cataract occurred in 22 eyes, glaucoma in 17 eyes, and cystoid macular oedema in 10 eyes. Diabetic retinopathy was detected in 38 (65.5%) eyes (29 non-proliferative including 6 with clinically significant macular oedema, and 9 proliferative). Progression of diabetic retinopathy to proliferative stage occurred in 7 eyes of 4 patients over a mean duration of 4.4 years. In 10 patients with active uveitis the mean HbA1c was 80 mmol/mol [9.5%], (range 49-137 [6.6-14.7]), and 67 mmol/mol [8.3%] (range 46-105 [6.4-11.8]) when the uveitis was quiescent, p = 0.01. Better glycaemic control was required in 10 patients during episodes of uveitis.

**Conclusions:**

Patients with DM who develop uveitis may have a high complication rate, reduced vision and poor glycaemic control. Checking blood glucose during episodes of uveitis is important.

## Background

Diabetic retinopathy is the third commonest cause of blindness in the Western world, with a 20% prevalence of blindness in the middle age group [1]. The incidence of diabetes mellitus (DM) is increasing rapidly in developed nations, and in the UK the number of people diagnosed has increased from 1.4 to 2.6 million since 1996 [2].

Uveitis and DM are individually potential sight threatening conditions. Uveitis may be idiopathic, associated with a range of systemic diseases, or caused by an infectious agent leading to variable intervals and degrees of vision impairment [3]. It is the fifth commonest cause of blindness in the middle age group with a prevalence of 10% in the developed world [1]. As both DM and uveitis predominantly occur in patients of working age, vision loss can have a major socioeconomic impact.

To our knowledge there is no study describing the clinical course of the diabetes and DM related eye changes in patients who develop uveitis with pre-existing DM. As both uveitis and DM disrupt blood-ocular barriers, we also wished to know the visual impact of these co-existing conditions. We acknowledge the limitations of the study as there was no control group and not all patients had HbA1C values.

## Patients and methods

This was a retrospective, longitudinal, case note study of patients attending the specialist Uveitis clinics at the Birmingham and Midland Eye Centre, a tertiary referral centre in the United Kingdom. Patients presenting with their first attack of uveitis from any cause who had pre-existing DM were included. Where appropriate, patients were investigated for any underlying systemic disease or infective cause. This included a full laboratory workup with full blood count, erythrocyte sedimentation rate, urea and electrolytes, liver function tests, C-reactive protein, angiotensin converting enzyme, anti-nuclear antibody, anti-neutrophil cytoplasmic antibody, syphilis serology and a chest x-ray. Additional investigations, such as toxoplasma antibodies and Mantoux test were undertaken where clinically indicated. Classification and grading of uveitis was undertaken according to the Standardization of Uveitis Nomenclature (SUN) Working Group classification [4]. The clinical course of the uveitis, frequency of recurrence and treatment undertaken were documented. The type of DM, DM treatment, changes in the DM treatment, types of diabetic retinopathy and maculopathy and its course and treatment were noted. Visual acuities and causes for poor vision were recorded. Where available HbA1c values throughout the clinical course were documented and compared with either active or quiescent uveitis.

## Results

A total of 58 eyes of 36 patients were included. Apart from 1 patient who was diagnosed with DM at presentation and had bilateral fibrinous uveitis and proliferative diabetic retinopathy all other patients were known to have pre-existing DM. The mean age (±SD) at the onset of DM was 48.6 (±13.9) years (range: 8-78 years), and the age at onset of uveitis was 55.4(±13.9) years (range: 33-82 years). The mean period (±SD) between the onset of DM and uveitis was 6.8 (±8.3) years (range: 0-31 years). The mean (±SD) follow up period was 4.4(±4.5) years (range: 1-18 years). There was an equal gender distribution; 17 patients were South Asian, 10 Caucasian and 9 African-Caribbean. The uveitis was bilateral in 22 patients and unilateral in 14 patients. There were 35 patients with Type 2 DM and 1 with Type 1 DM. Diabetic treatment at first presentation with uveitis comprised diet alone in 2 patients, oral hypoglycaemic agents (OHA) in 21 patients, and insulin in 13 patients (demographic data is summarised in Table 1). Impairment of glycaemic control had occurred in 10 patients with uveitis. 2 patients on diet control were started on OHAs, 2 patients on OHA had another agent added to their treatment, 1 patient who had stopped using OHA had to be restarted on it, 2 patients on OHA had to be started on insulin and 3 patients on insulin had to have their insulin dose increased to achieve better glycaemic control.

**Table 1:** Demographic data

**Parameter**	**Number**
Patients	36
Eyes	58
Gender	18 male, 18 female
**Race**	
Caucasian	10
South Asian	17
African-Caribbean	9
**Uveitis**	
Mean age of onset (years)	55
Laterality	14 unilateral, 22 bilateral
Anterior	32 eyes (19 patients)
Panuveitis	19 eyes (12 patients)
Intermediate	7 eyes (5 patients)
**Diabetes**	
Mean age of onset (years)	49
Type 1	1
Type 2	35

The anatomical type of uveitis was anterior in 32 eyes (19 patients), panuveitis in 19 eyes (12 patients), and intermediate in 7 eyes (5 patients). No patient had posterior uveitis. There were 32 patients (52 eyes) with non-infectious uveitis, and 4 patients (6 eyes) with an infectious cause. Of these 32 patients, 3 patients (3 eyes) had Fuchs’ heterochromic cyclitis, 2 patients (4 eyes) had sarcoidosis, and 2 patients (2 eyes) had HLA-B27 related uveitis. In 4 patients (6 eyes), it was presumed that the uveitis was a direct result of uncontrolled DM. These patients presented with severe uveitis, had high glycosylated haemoglobin, while other investigations to establish systemic associations were negative. Over the follow-up period, better glycaemic control was associated with improvement/resolution of the uveitis. The infectious causes in this cohort of patients were tuberculosis 2 patients (4 eyes), syphilis 1 patient (1 eye) and toxoplasma 1 patient (1 eye). Uveitis was acute in 14 patients, recurrent in 10 patients and chronic in 12 patients. The type of uveitis, disease association/syndromes are shown in Table 2.

**Table 2:** Uveitis disease associations/syndromes

**Clinical classification**	**Diagnosis**	**Number of eyes (patients)**
**Infectious**	Tuberculosis	4 (2)
	Syphilis	1 (1)
	Toxoplasmosis	1 (1)
**Non-infectious**	Sarcoidosis	4 (2)
	Fuchs’ heterochromic cyclitis	3 (3)
	HLA-B27 related	2 (2)
	Presumed diabetes mellitus	6 (4)
	Idiopathic	37 (21)

Presenting Snellen visual acuity was 6/12 or better in 26 eyes (45%), 6/18-6/60 in 19 eyes (33%) and worse than 6/60 in 13 (22%) eyes. Anterior chamber (AC) showed 3-4+ cellular reaction in 17 eyes (29%), 3-4+ flare in 19 eyes (32.7%), fibrin in 12 eyes (20.7%), and hypopyon in 3 eyes (5.2%). Macular status could not be assessed at presentation in some patients due to hazy media such as corneal oedema, small pupil, fibrin, cataract and vitreous haze.

The average number (±SD) of uveitic episodes in these patients was 3 (±2.2), range: 1-7 over the follow-up period. Cataract of varying degrees was seen in 22 eyes of 15 patients, posterior synechiae in 29 eyes, cystoid macular oedema (CMO) was present in 10 eyes of 8 patients. Raised intraocular pressure (IOP) requiring treatment was found in 17 eyes of 11 patients. One eye (one patient) developed iris bombé. Uveal effusion was noted in 1 eye that resolved with an improvement in the uveitis.

Diabetic retinopathy was noted in 38 eyes of 24 patients. Non-proliferative diabetic retinopathy was seen in 29 eyes of 19 patients. Proliferative diabetic retinopathy was seen in 9 eyes of 5 patients including vitreous haemorrhage in 3 eyes. All 9 eyes underwent panretinal laser photocoagulation. Apart from 1 patient who developed asymmetric diabetic retinopathy all other 23 patients had symmetric diabetic retinopathy. The patient with asymmetric diabetic retinopathy presented with unilateral panuveitis. Over 3 months the uveitic eye progressed to proliferative diabetic retinopathy while the non-uveitic eye remained at the non-proliferative stage. Clinically significant macular oedema developed in 6 eyes of 5 patients. These patients had non-proliferative diabetic retinopathy and received macular laser treatment. Fluorescein angiography and optical coherence tomography were not undertaken on a routine basis and was only available for some of the patients where clinically indicated such as in macular oedema. Some of the patients were seen in the period in which OCT was not routinely undertaken.

After a mean follow up period of 4.4 years, progression from non-proliferative to proliferative stage was noted in 7/38 (18.4%) eyes (4/24 patients, 16.7%) with diabetic retinopathy.

All patients received topical steroids and a dilating agent during the uveitic episode. Throughout the follow up period regional corticosteroid injections were required in 10 eyes (9 patients). Pulsed intravenous methylprednisolone (1 g/kg per day for 3 days) was given to 1 patient, 9 patients received systemic corticosteroids and 2 patients received systemic immunosuppressive drugs (1 azathioprine, 1 cyclosporine). Of these 9 patients who received systemic corticosteroids only 1 patient on long term 5 mg oral prednisolone had progression of non-proliferative to proliferative DR and 2 patients on short course of systemic corticosteroids needed better glycaemic control. In the patients with an infectious aetiology, 1 patient received a systemic anti-parasitic agent for clinically presumed toxoplasma uveitis, both patients with pulmonary tuberculosis were started on anti-tuberculosis therapy, and the patient with syphilis received intramuscular penicillin.

In those patients needing treatment for raised intraocular pressure, 1 patient (2 eyes) was given intravenous and oral acetazolamide with topical anti-glaucoma medications, and 10 patients (15 eyes) received only topical anti-glaucoma medications. Cataract surgery was performed in 19 eyes (13 patients), diagnostic vitreous biopsy was undertaken in 1 eye (1 patient), 2 eyes (2 patients) had vitrectomy for vitreous haemorrhage secondary to proliferative diabetic retinopathy, and 1 eye (1 patient) with iris bombé had a surgical peripheral iridectomy followed by trabeculectomy at a later stage for uncontrolled IOP.

HbA1c values were available in 10 patients during both active inflammatory stage and during the quiescent period (Table 3). The mean (±SD) HbA1c of 80 mmol/mol [9.5%] (±27 [2.4]) during active uveitis was statistically significantly higher than the mean (±SD) HbA1c of 67 mmol/mol [8.3%] (±27 [2.4]) during inactive uveitis, p = 0.01 (paired *t*-test). During the course of uveitis serial HbA1c values were available in 18 patients in the active period and in 20 patients in the quiescent period. Active period mean (±SD) was 75 mmol/mol [9.0%] (±26 [2.3]) and quiescent period mean (±SD) value was 61 mmol/mol [7.7%] (±16 [1.5]).

**Table 3:** HbA1c levels in active and quiescent periods of uveitis in 10 patients

**HbA1c levels in active period IFCC units - mmol/mol (DCCT units - %)**	**HbA1c levels in quiescent period IFCC units - mmol/mol (DCCT units - %)**
110 (12.2)	78 (9.3)
137 (14.7)	105 (11.8)
60 (7.6)	62 (7.8)
65 (8.06)	61 (7.75)
70 (8.6)	54 (7.1)
49 (6.6)	46 (6.4)
61 (7.7)	63 (7.9)
95 (10.8)	79 (9.4)
81 (9.6)	63 (7.9)
75 (9.0)	62 (7.8)

**Figure 1:**
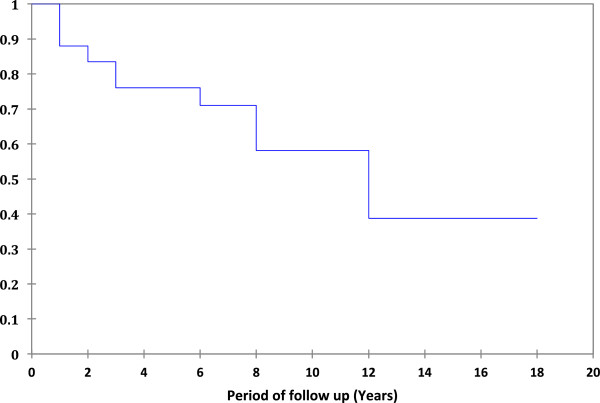
Kaplan-Meier survival curve for eyes with vision better than 6/18.

Figure 1 shows a Kaplan Meier survival curve for eyes with vision of better than 6/18. A visual acuity of 6/18 or worse was documented in 17 eyes (29%) of 15 patients. This was considered uveitis related in 9 eyes (9 patients). Persistent CMO was seen in 4 eyes (4 patients). The patients who had persistent CMO did not have any evidence of diabetic retinopathy; hence it was thought to be due to uveitis. Three eyes (3 patients) had macular epiretinal membrane/scar, 1 eye had vitreous debris and 1 eye had persistent vitreous inflammation. Poor vision appeared diabetic retinopathy related in 4 eyes of 3 patients. In 3 eyes (2 patients) the cause was diabetic maculopathy and in 1 eye was due to vitreous haemorrhage. Other causes of poor vision in this group of patients was central retinal vein occlusion in 1 eye, advanced cataract and glaucoma in in each eye of 1 patient who refused treatment for both these conditions, and age related macular degeneration in 1 eye. The causes of reduced vision are summarised in Table 4.

**Table 4:** Causes of reduced vision

**Uveitis related**	**Number of eyes (patients)**
Persistent CMO	4 (4)
Macular scar/ERM	3 (3)
Vitreous debris/inflammation	2 (2)
**Diabetic retinopathy related**	
Maculopathy	3 (2)
Vitreous haemorrhage	1 (1)
**Others**	
CRVO	1 (1)
Cataract with glaucoma	2 (1)
AMD	1 (1)

*CMO* Cystoid Macular Oedema, *ERM* Epiretinal Membrane, *CRVO* Central Retinal Vein Occlusion.

## Discussion

This longitudinal study aimed to investigate the clinical course when uveitis from any cause and DM has coexisted and its impact on visual outcome. It was not our aim to compare uveitis patients with and without DM. We also wished to know if uveitis had any bearing on blood sugar control. The relationship between DM and uveitis has been proposed by a number of reports [5-10] but to our knowledge this is the first study in a cohort of patients with DM in whom the clinical course of uveitis, relationship to blood sugar control, progression of diabetic retinopathy and visual outcome have been studied.

Blindness due to diabetic retinopathy and uveitis are both potentially treatable and among the top five commonest causes of blindness in the middle-aged population [1]. In our cohort of middle-aged patients 42% (15 patients, 17 eyes) had final visual acuity worse than 6/18. In 9 eyes (53%) the poor visual acuity was thought to be uveitis related, with four of these eyes having persistent CMO. Coexistence of these two conditions appears to have a higher rate of poor visual acuity.

Interestingly the majority of our cohort (26 patients, 72%) were from ethnic minorities (South Asian and African-Caribbean origin) that probably reflects the population served by the Birmingham and Midland Eye Centre although it appears much higher than the average population of ethnic minorities of 29.6% in Birmingham compared to 9.1% in England as per the 2001 census. There is recent evidence suggestive of a high prevalence of DM in ethnic minorities in the UK [11] that may contribute to the increased number of this cohort of patients in our study.

We have previously published the presenting features of uveitis in most of these patients [12]. Our study suggests that patients with pre-existing DM presenting with uveitis may exhibit increased intraocular inflammation that does not appear to be related to the type of uveitis and subsequently had on average three recurrences. There was evidence of high degrees of AC flare, dense fibrin and cellular reaction. This severe fibrinous reaction was likely to be responsible for the large number of eyes with posterior synechiae (50%) and the eyes with raised IOP (29%). In four patients the uveitis was likely to be related to DM as investigations failed to reveal any underlying cause, the uveitis resolved with better glycaemic control, and all had a severe fibrinous reaction in the AC in line with previous reports [5-10,12]. It is interesting that severe inflammation was also noted in the eyes where DM was not suspected as a cause of the uveitis. It is likely that the severe inflammation was related to the additional blood-ocular barrier breakdown and/or ocular ischaemia from pre-existing DM, particularly as there was mean of almost seven years between diagnosis of DM and onset of uveitis, and some patients already had evidence of diabetic retinopathy.

Cataract has been reported as a cause of visual loss in 17.7% of patients with uveitis [3]. DM has been implicated as a risk factor in the causation of cataract [13-15]. Our cohort had high incidence of cataract (38%) that may be due to a combination of uveitis and DM. During the course of uveitis 12 patients (33%) needed systemic/regional corticosteroids and/or immunosuppressive agents to gain control of inflammation. This highlights the severity of inflammation and the requirement to intervene with second or third line treatment strategies to achieve control of the inflammation.

In a study of over 10,000 patients with DM in the UK the prevalence of diabetic retinopathy has been reported to be 16.5% [16,17]. Our cohort had diabetic retinopathy in 24 patients (66.7%), which is four times higher. The UK Prospective Diabetes Study showed that people with improved glucose control reduced the requirement of laser treatment of the eye by a quarter [18]. Progression of diabetic retinopathy to the proliferative stage over a 4-year follow up in WESDR study in 1075 patients was reported to be 4.7% [19]. In our study 18.4% of patients went on to develop proliferative diabetic retinopathy over an average of 4.36 years. The co-existing uveitis could trigger mechanisms for progression of diabetic retinopathy amongst other factors. However we were unable to identify other main risk factors, such as hypertension and hyperlipidaemia to have a contributory role in our cohort. It was interesting to note rapid progression of non-proliferative diabetic retinopathy to proliferative in one eye of a patient with panuveitis in that eye. There are a number of reports in the literature suggesting either a causative or a protective role of uveitis in relation to proliferative diabetic retinopathy [20-23]. In the current literature 6 uveitic eyes progressed to PDR in patients with DM whereas two eyes did not progress. This suggests a possible role of worsening diabetic retinopathy in patients with uveitis.

We have previously shown that HbA1c is raised in DM patients at presentation with uveitis [12]. We attempted to correlate glycaemic control with episodes of uveitis. HbA1c values were available in 10 patients during the active uveitic phase as well as in the quiescent phase. All patients had a value higher than the recommended 44 mmol/mol (6.2%) in the active phase as compared to the quiescent phase that was statistically significant. This suggests that poor glycaemic control may be a trigger for the reactivation of the uveitis. Overall, HbA1c values were available in 18 patients during the active phase and in 20 patients in the inactive phase. The mean value during the active phase was much higher than the quiescent phase. A limitation of this study is its retrospective nature and regular glycosylated haemoglobin values were not available in all patients.

During the course of uveitis 10 patients needed a change in their DM treatment to gain better glycaemic control that was thought to help in control of the inflammation. This highlights the need to check the blood glucose levels and HbA1c levels in patients with DM who get uveitis, irrespective of aetiology. Treatment with systemic corticosteroid therapy can influence the glycaemic control. Only one of these patients was on low dose of 5 mg oral prednisolone and 2 received short course of systemic steroids. They all required multidisciplinary input to gain better blood sugar control. Stabilization of blood sugars seemed to help in controlling the inflammation.

## Conclusions

Uveitis occurring in patients with pre-existing diabetes can be associated with numerous ocular complications and recurrences. Macular involvement related to both the uveitis and the diabetes appears to be the main cause of reduced vision. Better control of DM with treatment may result in better control of inflammation as seen in some of our patients. Where uveitis and diabetes co-exist, ophthalmologists and diabetic physicians should be aware that glycaemic control might not be optimal. Monitoring glycaemic control in all diabetics presenting with uveitis should be mandatory.

## Abbreviations

AC: Anterior chamber; CMO: Cystoid macular oedema; DM: Diabetes mellitus; IOP: Intraocular pressure; Kg: Kilogram; OHA: Oral hypoglycaemic agents; SD: Standard deviation; SUN: Standardization of Uveitis Nomenclature; WESDR: Wisconsin Epidemiologic Study of Diabetic Retinopathy.

## Competing interests

The authors declare that they have no competing interests.

## Authors’ contributions

KSO, RRS, PIM, PS all made substantial contributions to conception and design of the study. Data acquisition was mainly undertaken by KSO and RRS, and analysis of data by KSO, RRS, PIM, PS. KSO, PIM and PS were all involved in drafting the manuscript and KSO, RRS, PIM, PS were all involved in revising it critically. All authors read and approved the final manuscript.
